# Investigation of the Prevalence of Diminished Ovarian Reserve in Korean Women of Reproductive Age

**DOI:** 10.3390/jcm12155099

**Published:** 2023-08-03

**Authors:** Rihwa Choi, Wonseo Park, Gayoung Chun, Sang Gon Lee, Eun Hee Lee

**Affiliations:** 1 Department of Laboratory Medicine, Green Cross Laboratories, Yongin 16924, Republic of Korea; pirate0720@naver.com; 2 Department of Laboratory Medicine and Genetics, Samsung Medical Center, Sungkyunkwan University School of Medicine, Seoul 06351, Republic of Korea; 3 Infectious Disease Research Center, Green Cross Laboratories, Yongin 16924, Republic of Korea; realdenma@gclabs.co.kr (W.P.); forjund@gclabs.co.kr (G.C.); 4 Green Cross Laboratories, Yongin 16924, Republic of Korea

**Keywords:** infertility, ovarian reserve, aging, anti-Müllerian hormone, public health, Korea

## Abstract

Diminished ovarian reserve can be assessed biochemically using serum anti-Müllerian hormone (AMH) and follicle-stimulating hormone (FSH) tests. This study aimed to evaluate the prevalence of diminished ovarian reserve in a large population of reproductive-aged women by age and geographic region in Korea using different cutoffs of serum AMH and FSH levels. In 2022, 13,351 women underwent both AMH and FSH tests. The prevalence of diminished ovarian reserve increased markedly with age. Although cutoffs for AMH and FSH levels are different in USA and Korean guidelines, the overall prevalence of diminished ovarian reserve was comparable. The maximum prevalence was 3.8%, 6.0%, 11.0%, 28.6%, 69.3%, and 95.0% in women aged 20–24, 25–29, 30–34, 35–39, 40–44, and 45–49 years, respectively. The overall prevalence and age-adjusted prevalence of diminished ovarian reserve were 37.2% and 38.4%, respectively. Women who had only increased serum FSH without decreased AMH represented 1.1% (by Korean guidelines) and 2.5% (by USA guidelines) of all women. Serum AMH and FSH tests were underutilized on Jeju Island. The results of this study provide basic knowledge about diminished ovarian reserve for use in infertility support programs and the field of maternal aging.

## 1. Introduction

Maternal aging and infertility are regional as well as global concerns [1]. A recent report by the World Health Organization (WHO) in 2022 stated a global infertility lifetime prevalence estimate of 17.5%, and among all regions, the Western Pacific Region including Korea showed the highest prevalence of lifetime infertility (23.2%) [1]. Anti-Müllerian hormone (AMH) and follicle-stimulating hormone (FSH) are essential hormones that have considerable roles in female reproductive health [2,3,4]. AMH is produced by granulosa cells in small growing ovarian follicles and serves as a marker of ovarian reserve, which declines with age and is associated with the size of the follicular pool and the number and quality of eggs remaining in the ovaries [5,6]. FSH is produced by the pituitary gland and stimulates the growth and maturation of ovarian follicles, which contain eggs [2,3,4]. FSH levels rise with increasing age and diminished ovarian reserve, indicating that greater FSH stimulus is required to drive folliculogenesis [5,6,7]. Therefore, decreased serum AMH and increased FSH concentrations can be used to assess ovarian reserve [4,7]. The reproductive potential of the ovaries represents potential fertilization and can be predicted using both ultrasound imaging tests and biochemical measurements of serum AMH and FSH [3,8]. Serum AMH fluctuates less by menstrual cycle, and basal serum FSH is a specific but not sensitive test for diminished ovarian reserve; both tests are used to predict future response to ovarian stimulation for in vitro fertilization (IVF) [3,6,7].

There are clinical guidelines for predicting ovarian response and diagnosis of diminished ovarian reserve globally. In Europe, the European Society of Human Reproduction and Embryology (ESHRE) published a guideline stating that either antral follicle count (AFC) or AMH is recommended over other ovarian reserve tests using the Bologna criterion of AMH < 1.1 ng/mL to define a patient as a poor ovarian responder [9]. The American College of Obstetricians and Gynecologists (ACOG) and the American Society for Reproductive Medicine (ASRM) consider AMH < 1 ng/mL and FSH > 10 mIU/mL to be indicative of diminished ovarian reserve [3,4,7].

Meanwhile, the serum AMH test was introduced in Korea in December 2016. This test is reimbursed by the Korean government (Health Insurance Review and Assessment Service, HIRA) based on the following specific criteria: performed to investigate infertility, before or after ovarian surgery, before or after chemotherapy and/or radiation therapy, and poor ovarian stimulation response since December 2019. The Maternal and Child Health Act of Korea has implemented a national support program for married couples with infertility, and the criteria for diminished ovarian reserve for the supporting program are serum AMH ≤ 1.0 ng/mL and serum FSH ≥ 12.0 mIU/mL [10]. These variations in definitions in inclusion/exclusion criteria in infertility studies have resulted in challenges in research on maternal aging and infertility [1].

Previous studies on AMH in Korea have involved a limited number of subjects, used different cutoffs for diminished ovarian reserve, or focused on continuous variables to define reference intervals [11,12,13,14,15]. Additionally, previous studies on serum AMH, FSH, and IVF have been conducted using data from women who visited university hospitals in Korea [11,14]. The prevalence of diminished ovarian reserve based on serum AMH and FSH tests in a larger Korean population has not been extensively studied, and there is a lack of recent information about its prevalence as a possible indication for IVF and/or national infertility support programs, particularly in the context of maternal aging.

Laboratory information system-based data analysis about the specimen prevalence of specific analytes and their result combinations and/or patterns can give important implications for the quality management of a clinical laboratory, with appropriate utilization of tests and epidemiological information [16,17]. Therefore, this study aimed to evaluate the prevalence of diminished ovarian reserve in a large population of reproductive-aged women by age and geographic region in Korea using different cutoffs of serum AMH and FSH levels. This basic knowledge of the prevalence of diminished ovarian reserve based on biochemical data will help public health agencies to plan support for married Korean couples with infertility issues using IVF.

## 2. Materials and Methods

### 2.1. Study Subjects

We retrospectively reviewed laboratory data for results of serum AMH and FSH tests performed in Korean women of reproductive age (20.0 to 49.0 years) through the laboratory information system of Green Cross Laboratories between 1 January 2022 and 31 December 2022, considering the legal age in Korea (women aged > 20.0 years) and the upper limit of WHO’s women of reproductive age criteria (women aged 49 years) [18]. Green Cross Laboratories is a referral laboratory that provides specimen analysis for serum AMH and FSH tests as requested by local clinics and hospitals in Korea. Data for serum AMH and FSH levels were anonymized before analysis. Records missing data on age or sex were excluded. Because the aim of this study was to investigate the prevalence of diminished ovarian reserve, repeated measurements in the same individuals were excluded. Data from those who had serum AMH or serum FSH only were also excluded.

### 2.2. Definitions

Serum AMH and FSH cutoffs for diminished ovarian reserve were categorized using two different guidelines. The first one, based on ACOG and ASRM recommendations, uses serum AMH < 1.0 ng/mL and serum FSH > 10.0 mIU/mL to define diminished ovarian reserve [3,4]. The second one, based on Korean guidelines for infertility support programs, uses serum AMH ≤ 1.0 ng/mL or serum FSH ≥ 12.0 mIU/mL to define diminished ovarian reserve [10]. Serum AMH and FSH results and the prevalence of diminished ovarian reserve were investigated by age group as follows: 20–24, 25–29, 30–34, 35–39, 40–44, and 45–49 years.

To create maps of the prevalence of diminished ovarian reserve by geographic region, we grouped the administrative districts of Korea into six categories based on geographic proximity and regional codes available in the laboratory information system [19]. The number of total Korean women by geographic region was collected from a public database of the Korean Statistical Information Service (KOSIS) for the resident registry population (https://kosis.kr/index/index.do; accessed on 10 April 2023) [20].

### 2.3. Analytical Methods

Serum AMH and FSH were measured using Elecsys AMH Plus assay kits (Roche Diagnostics GmbH, Mannheim, Germany) and Elecsys FSH (Roche Holding AG, Basel, Switzerland) on Cobas 8000 e801 analyzers (Roche Diagnostics GmbH, Mannheim, Germany). The analytical measurement range (AMR) for serum AMH ranged from 0.01 to 23.00 ng/mL, and the AMR for serum FSH ranged from 0.3 to 200 mIU/mL. The accuracy of the serum AMH and FSH assays was ensured through participation in a quality management program and proficiency testing provided by the Korean Association of External Quality Assessment Service and College of American Pathologists in the United States: AMH (anti-Müllerian hormone), Y (sex hormones), LN8 (reproductive endocrinology calibration verification/linearity), and ABS (accuracy-based testosterone and estradiol) surveys [15].

### 2.4. Statistical Analysis

Statistical analyses for continuous variables (age, AMH, and FSH levels) were executed using non-parametric methods when appropriate. The prevalence of diminished ovarian reserve was investigated based on serum AMH and/or serum FSH criteria using two different guidelines during the study period. The prevalence of diminished ovarian reserve was examined by group based on age. The chi-squared test was used to investigate the differences in the prevalence of diminished ovarian reserve based on criteria, sex, age group, and geographic regions. Statistical analysis was performed using MedCalc Statistical Software version 20.216 (MedCalc Software Ltd., Ostend, Belgium; https://www.medcalc.org, accessed on 10 April 2023). A *p* value was considered significant at the 0.05 level. The prevalence of diminished ovarian reserve by geographic region was adjusted by the number of total women in the resident registry population (https://kosis.kr/index/index.do; accessed on 10 April 2023). Maps of the prevalence of diminished ovarian reserve and the prevalence of women managed for infertility were created using R software (version 4.2.2; http://www.R-project.org/; accessed on 10 April 2023).

## 3. Results

**Figure 1:**
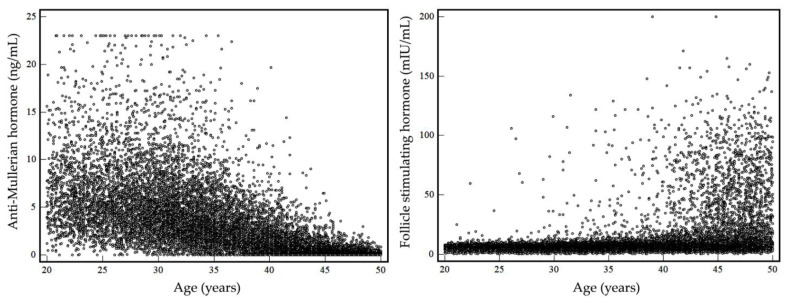
Serum AMH and FSH levels with increasing age.

During the 1-year study period, a total of 13,588 specimens with both serum AMH and FSH results from 13,351 women were screened. After excluding 237 repeated measurements, 13,351 test results were included in the investigation of the prevalence of diminished ovarian reserve. Among these, no missing data for age and sex were observed. A total of 13,351 Korean women with a median age of 35.9 years (interquartile range aged 30.0 to 43.2) had been tested for serum AMH and FSH levels, simultaneously. The number of women was highest in the group aged 30–34 years (21.5%), followed by 40–44 years (18.4%). The smallest group included women aged 20–24 years (9.6%). Numbers of women by age group and their serum AMH and FSH levels are summarized in Table 1. Data for age and serum AMH and FSH levels by age group were not normally distributed. Median serum AMH levels decreased with age and median serum FSH levels increased with age. Serum AMH and FSH levels with increasing age are presented in Figure 1.

**Figure 2:**
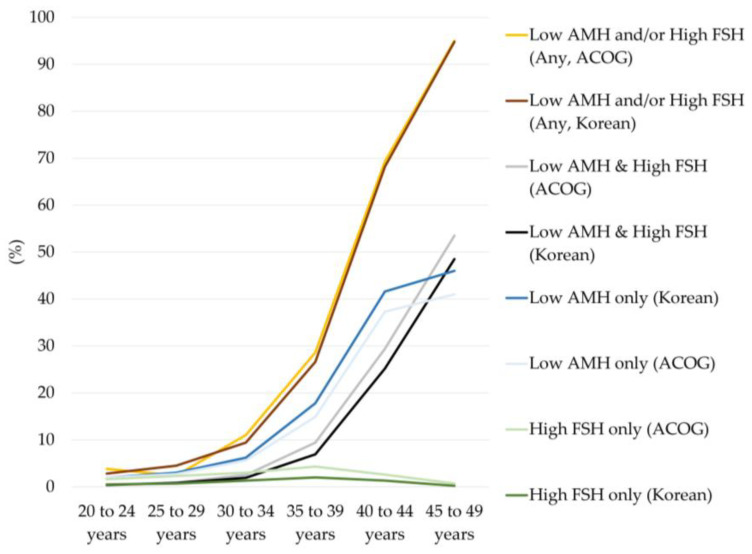
Prevalence of diminished ovarian reserve by age group and criteria. ACOG means criteria for defining diminished ovarian reserve used by the American College of Obstetricians and Gynecologists and the American Society for Reproductive Medicine (ASRM, serum AMH < 1.0 ng/mL or serum FSH > 10.0 mIU/mL). Korean means criteria used by the Korean guidelines for the infertility support program (serum AMH ≤ 1.0 ng/mL or serum FSH ≥ 12.0 mIU/mL) to define diminished ovarian reserve. Lighter colors represent results according to the ACOG and ASRM criteria, and darker colors represent results as defined by the Korean guidelines for an infertility support program.

The prevalence of diminished ovarian reserve based on different cutoffs for serum AMH and FSH levels using different guidelines is summarized in Table 2 and Figure 2.

The prevalence of diminished ovarian reserve markedly increased with age. Among all women, the prevalence of diminished ovarian reserve based on any of the met AMH or FSH criteria was 38.5% using ACOG and ASRM cutoffs (AMH < 1.0 ng/mL or FSH > 10.0 mIU/mL) and 37.2% using the Korean guideline cutoff (AMH ≤ 1.0 ng/mL or FSH ≥ 12.0 mIU/mL). The prevalence of diminished ovarian reserve based only on met FSH criteria was the lowest among those of all criteria, and overall prevalence was 2.5% and 1.1% using the ACOG and ASRM cutoffs and the cutoff of the Korean guidelines for infertility support programs, respectively. About half of the women had met the criteria of either decreased AMH only or both decreased AMH and increased FSH levels.

In women under 30 years of age, the prevalence of diminished ovarian reserve was <5.0% for all criteria. However, the prevalence increased up to about 10.0% in women aged 30–34 years, >25.0% in women aged 35–39 years, and more than 2/3 in women aged 40–44 years, and most women had diminished ovarian reserve at 45–49 years (95.0%). The prevalence of diminished ovarian reserve was higher for the Korean guidelines than for ACOG and ASRM when only AMH criteria were applied (maximal difference 5.0%). However, the prevalence of patients was lower when using the Korean guidelines than when using ACOG and ASRM when both AMH and FSH result criteria were met (maximal difference 5.0%). This led to the result that the overall prevalence of diminished ovarian reserve was comparable between the ACOG and ASRM guidelines and Korean guidelines for infertility support programs when any of the AMH and FSH results criteria were met.

**Figure 3:**
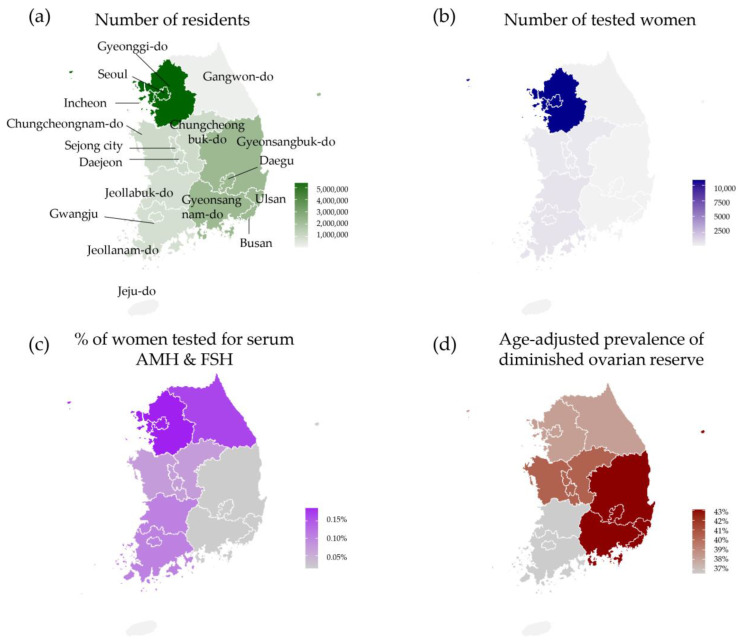
Number and prevalence of cases of diminished ovarian reserve as defined by the Korean guidelines for the infertility support program (serum AMH ≤ 1.0 ng/mL or serum FSH ≥ 12.0 mIU/mL) by geographic region. (**a**) Number of women living in Korea [20]. (**b**) Number of women tested for serum AMH and FSH through laboratory information system. (**c**) Proportions of women tested for serum AMH and FSH by geographic region. (**d**) Age-adjusted prevalence of diminished ovarian reserve by geographic region.

Test utilization of serum AMH and FSH tests and the prevalence of diminished ovarian results by geographic region are presented as a map. The prevalence of diminished ovarian reserve according to both AMH and FSH levels by geographic region in Korea is presented in Table 3 and Figure 3. The differences in the prevalence of diminished ovarian reserve between regions were determined to be statistically significant (*p* < 0.0001).

In Korea, the majority of women live in the Seoul, Gyeonggi-do, and Incheon areas (54.9% of all women). The proportion of women tested for serum AMH and FSH in this region was 81.5% of all study subjects. No women living in Jeju-do (Jeju Island) were tested for serum AMH and FSH. In this study population, the number of women tested in Gyeongsang province was less than the number of women tested in the other provinces. Age-adjusted prevalence of diminished ovarian reserve ranged from 38.2% (Gangwon-do) to 43.1% (Gyeongsang province), with an overall rate of 38.4%.

## 4. Discussion

This study investigated the prevalence of biochemically defined diminished ovarian reserve in over 10,000 Korean women of reproductive age using their serum AMH and FSH levels. Although public databases in Korea report the number of patients managed for infertility, there is no information on diminished ovarian reserve, which is a knowledge gap filled through this study.

The prevalence of diminished ovarian reserve observed in this study was evaluated based on biochemical results, and it was higher (age-adjusted prevalence 38.4% with AMH ≤ 1.0 ng/mL or FSH ≥ 12 mIU/mL) than that in previous studies conducted in Western populations (ranging from 19% to 26% in different cohorts) using the same FSH cutoff (≥12 mIU/mL) [7,21]. The mean age of the study population in this study was 36.2 years (standard deviation 7.97), which was slightly higher than the maximum mean age of 35.6 years (standard deviation 4.8) reported in previous studies [7,21]. This could potentially impact the prevalence of diminished ovarian reserve defined by biochemical markers, given that the prevalence of diminished ovarian reserve tends to increase significantly with age [1,2]. The prevalence of diminished ovarian reserve in Western populations (19 to 26%) was comparable with the prevalence of women of 35–39 years of age in the present study cohort (26.6%) [7,21].

Here, the prevalence of diminished ovarian reserve was markedly increased in women aged more than 30 years old. According to the Healthcare BigData Hub of HIRA, the number of women aged 20–49 years and managed for infertility (using the code of Korean Standard Classification of Diseases N97 female infertility) gradually increased from 158,490 in 2017 to 171,190 in 2021 [22,23]. Among them, the largest population was women aged 30–39 years (70.7–72.6%). According to the public database of the Korean Statistical Information Service (KOSIS), the mean age of first delivery has gradually increased from 31.6 years in 2017 to 32.6 years in 2021 [20]. The Korean government has a public program for financial support for families experiencing infertility to improve the birth rate [24,25,26]. Considering the increase in the prevalence of diminished ovarian reserve in women over 30 years and the gradually increasing median age of marriage and childbearing, a public health plan should focus on improving maternal and child health with consideration of maternal age. For example, in the USA, IVF has been suggested as a first-line treatment strategy in women older than 38 to 40 years because female fecundity declines with age [7]. National education programs regarding aging and diminished ovarian reserve for reproductive health and family planning including maternal aging and birth outcomes, such as maternal and child health, may be helpful for women with their family planning [21]. Monitoring the prevalence of diminished ovarian function using biochemical markers may be used as basic information for preparing treatment strategies to improve birth rates and maternal outcomes [16,17,21].

In this study, a small proportion of women was additionally identified as having diminished ovarian reserve based on their FSH level (overall 1.1% for Korean guideline of ≥12 mIU/mL and 2.5% for ACOG and ASMR guideline of FSH > 10.0 mIU/mL). Considering that ESHRE guidelines use only AMH as a biochemical predictor of diminished ovarian reserve, slightly more Korean women could be suspected to have diminished ovarian reserve compared to European women, and slightly fewer Korean women could be suspected to have diminished ovarian compared to women in America. However, diminished ovarian reserve should be approached with AFC and other clinical findings to avoid this effect. High FSH levels alone could be due to various factors, such as fluctuations during the menstrual cycle (early follicular phase), impending ovulation, early pregnancy, FSH-secreting ovarian cysts, and possible assay-specific interferences caused by medications, immunoglobulins, or rare genetic conditions affecting the binding site of antibody reaction in immunoassays [2,3,27]. These factors should be taken into account when interpreting cases with isolated high FSH levels. Future studies are needed to assess the clinical impacts of the differences in biomarkers, considering their association with physiological and analytical aspects, as well as cutoffs for these biomarkers in different regions.

The proportion of women tested for FSH and AMH varied among different geographic regions. In this study, the proportion of women requesting tests from the Seoul, Gyeonggi-do, and Incheon areas was the major population (81.5%), in agreement with information on the proportion of women who had been tested for D3730 serum AMH in the HIRA public database (70.0%) [23]. The smallest number of tests by geographic area occurred in Jeju-do (Jeju Island, none). In Gyeonsang province, only 2.5% of residents underwent serum AMH and FSH testing. The findings, indicating that 70.0–81.5% of AMH/FSH levels were recorded in Seoul/Gyeonggi-do/Incheon, despite only 50% of Korean women residing in those areas, suggest that the health behaviors of women in different geographic regions may influence the utilization of AMH/FSH tests and could be associated with differences in healthcare access capability [20,23,24]. This finding was comparable with the previous reports and data from HIRA regarding the limited healthcare access and test utilization of that area [20,28]. These disparities in utilization could significantly affect prevalence estimation and should be considered during the interpretation of study results.

A laboratory information system managing large numbers of patient results can provide important direct or indirect epidemiological information not only for the laboratory and region, but also on a nationwide scale [17]. According to the Healthcare BigData Hub of HIRA, about half (47.7%) of serum AMH tests in 2021 were performed in local clinics, followed by hospital-level medical institutions (20.3%), general hospitals (16.9%), and tertiary hospitals (including university hospitals, 14.1%) [23]. However, most clinical studies were performed on patients from university hospitals, limiting the generalization. Therefore, the prevalence of diminished ovarian reserve based on local clinics and hospitals is valuable information about a more general prevalence of women visiting local clinics and hospitals.

A limitation of the present study was the lack of clinical information associated with ovarian reserve, such as menstruation, antral follicle count on ultrasonography, medications, comorbidities, and a history of poor response to IVF stimulation [5,17,29,30]. Caution should be exercised when interpreting the prevalence identified in this study, as the study population comprised Korean women who had undergone testing for both serum AMH and FSH. These women might have had conditions such as infertility, previous ovarian surgery, chemotherapy, or poor response to stimulation, which could potentially lead to an overestimation of the prevalence in the general population [2,3]. The study period includes the severe acute respiratory syndrome coronavirus 2 (SARS-CoV-2) pandemic period, in which infection and/or immunization history may have affected reproductive function and hormonal changes and limited the generalizability of the present study’s results [30,31,32]. Also, the small number of tests requested from some geographic regions might affect the estimated prevalence of diminished ovarian reserve. However, our study findings may be generalizable to populations that access healthcare facilities, such as local clinics and hospitals, to undergo serum AMH and FSH tests in Korea. Considering that previous studies were based on data from subjects visiting university hospitals, which may have different characteristics, such as comorbidities and healthcare behaviors, compared with women visiting local obstetrics clinics and hospitals, this study has the strength of providing basic information on the prevalence of women in the Korean population with possible diminished ovarian reserve [17]. Future research is needed to explore population selection bias and its implications on the prevalence of diminished ovarian reserve, considering various factors associated with healthcare utilization and access across different geographic regions. Future studies with comprehensive clinical information, including about menstruation, history of IVF stimulation, ultrasonographic findings, and other comorbidities that might affect test results, are needed to clarify the utilization and implication of serum AMH and FSH tests and the number and prevalence of patients with diminished ovarian reserve.

The strengths of the present study include the large number of subjects studied to analyze diminished ovarian reserve prevalence in the Korean population, who might subsequently benefit from a national infertility support program [10]. The results of this study could help inform public health programs to improve maternal and child health in the Korean population. Furthermore, determining diminished ovarian reserve prevalence by age group could help women at greater risk who require the further support of infertility treatment. The results of this study could also help strengthen an understanding of biochemical tests to define diminished ovarian reserve in the Korean population.

## 5. Conclusions

In conclusion, this study investigated the prevalence of diminished ovarian reserve in a large number of Korean women based on serum AMH and FSH levels. The prevalence of diminished ovarian reserve increased significantly with age and more than doubled in women over 30 years old compared to those under 30 years old. These study results can help to strengthen the understanding of biochemical tests used to define diminished ovarian reserve within the Korean population. As the prevalence of diminished ovarian reserve increases in women over 30 years of age and the median ages of marriage and childbearing continue to rise, public health initiatives should prioritize improving maternal and child health based on maternal age.

## Figures and Tables

**Table 1:** Serum AMH and serum FSH test result distribution by age for 13,351 Korean women.

Variables	N	Min	2.5th	5th	10th	25th	Med	75th	90th	95th	97.5th	Max	Mean	SD
Age	13,351	20.0	21.7	23	25.2	30.0	35.9	43.2	47.1	48.4	49.0	49.9	36.2	7.97
Anti-Müllerian Hormone (ng/mL)														
20–24 years	1280	0.01	1.05	1.69	2.34	3.72	5.78	8.63	12.30	15.15	18.00	23.00	6.70	4.16
25–29 years	2049	0.01	0.75	1.21	1.77	3.23	5.13	7.88	11.60	14.61	17.20	23.00	6.10	4.20
30–34 years	2874	0.01	0.33	0.63	1.20	2.24	3.79	6.17	9.19	11.58	14.77	23.00	4.69	3.60
35–39 years	2269	0.01	0.01	0.12	0.36	1.03	2.14	3.76	5.79	7.31	9.44	23.00	2.79	2.60
40–44 years	2456	0.01	0.01	0.01	0.01	0.11	0.54	1.35	2.55	3.82	4.92	19.70	1.03	1.45
45–49 years	2423	0.01	0.01	0.01	0.01	0.01	0.08	0.30	0.75	1.05	1.52	5.70	0.26	0.45
Follicle-stimulating hormone (mIU/mL)														
20–24 years	1280	0.3	1.5	1.8	2.7	4.4	5.8	7.0	8.1	8.9	9.8	59.6	5.8	2.8
25–29 years	2049	0.3	1.5	2.1	2.8	4.4	5.8	7.1	8.3	9.3	10.5	116.0	6.1	5.4
30–34 years	2874	0.3	1.7	2.3	3.1	4.7	6.2	7.5	8.9	10.2	13.2	134.0	6.7	6.6
35–39 years	2269	0.3	1.8	2.3	2.9	4.7	6.4	8.2	11.3	17.4	41.1	200.0	8.7	13.1
40–44 years	2456	0.3	2.0	2.4	3.1	4.7	7.1	12.8	45.8	73.9	90.3	200.0	16.0	23.5
45–49 years	2423	0.3	0.0	2.7	3.6	5.8	11.5	37.5	74.5	93.6	106.0	165.0	26.0	29.9

Abbreviations: Max, maximum; Med, median; Min, minimum; SD, standard deviation.

**Table 2:** Prevalence of diminished ovarian reserve based on serum AMH and FSH levels using different categories.

Guide	Ovarian Reserve by Serum AMH and/or FSH	All Age (n = 13,351)		20–24 Years (n = 1280)		25–29 Years (n = 2049)		30–34 Years (n = 2874)		35–39 Years (n = 2269)		40–44 Years (n = 2456)		35–39 Years (n = 2423)	
		n	%	n	%	n	%	n	%	n	%	n	%	n	%
ACOG and ASRM	Normal (AMH ≥ 1.0 ng/mL and FSH ≤ 10.0 mIU/mL)	8213	61.5	1231	96.2	1927	94.0	2557	89.0	1621	71.4	755	30.7	122	5.0
	DOR by AMH only (AMH < 1.0 ng/mL and FSH ≤ 10.0 mIU/mL)	2487	18.6	24	1.9	56	2.7	160	5.6	337	14.9	917	37.3	993	41.0
	DOR by FSH only (AMH ≥ 1.0 ng/mL and FSH > 10.0 mIU/mL)	329	2.5	20	1.6	47	2.3	85	3.0	97	4.3	63	2.6	17	0.7
	DOR by both AMH and FSH (AMH < 1.0 ng/mL and FSH > 10.0 mIU/mL)	2322	17.4	5	0.4	19	0.9	72	2.5	214	9.4	721	29.4	1291	53.3
	DOR by any of AMH or FSH (AMH < 1.0 ng/mL or FSH > 10.0 mIU/mL)	5138	38.5	49	3.8	122	6.0	317	11.0	648	28.6	1701	69.3	2301	95.0
Korean	Normal (AMH > 1.0 ng/mL and FSH ≥ 12.0 mIU/mL)	8380	62.8	1244	97.2	1957	95.5	2605	90.6	1665	73.4	782	31.8	127	5.2
	DOR by AMH only (AMH ≤ 1.0 ng/mL and FSH < 12.0 mIU/mL)	2800	21.0	24	1.9	61	3.0	177	6.2	403	17.8	1021	41.6	1114	46.0
	DOR by FSH only (AMH > 1.0 ng/mL and FSH ≥ 12.0 mIU/mL)	142	1.1	7	0.5	15	0.7	36	1.3	45	2.0	33	1.3	6	0.2
	DOR by both AMH and FSH (AMH ≤ 1.0 ng/mL and FSH ≥ 12.0 mIU/mL)	2029	15.2	5	0.4	16	0.8	56	1.9	156	6.9	620	25.2	1176	48.5
	DOR by any of AMH or FSH (AMH ≤ 1.0 ng/mL or FSH ≥ 12.0 mIU/mL)	4971	37.2	36	2.8	92	4.5	269	9.4	604	26.6	1674	68.2	2296	94.8

Abbreviations: ACOG, American College of Obstetricians and Gynecologists; AMH, anti-Müllerian hormone; ASRM, American Society for Reproductive Medicine; DOR, diminished ovarian reserve; FSH, follicle-stimulating hormone; Korean, Korean guideline for infertility support program.

**Table 3:** Number and prevalence of cases of diminished ovarian reserve by age and geographic region.

Characteristics	No. of Tested Women	Diminished Ovarian Reserve ^1^			No. of Residents
		N	Prevalence (%)	Age-Adjusted Prevalence (%)	
Total	13,351	4971	37.2	38.4	10,273,563
20–24 years	1280	36	2.8		1,449,453
25–29 years	2049	92	4.5		1,651,173
30–34 years	2874	269	9.4		1,553,373
35–39 years	2269	604	26.6		1,650,151
40–44 years	2456	1674	68.2		1,956,066
45–49 years	2423	2296	94.8		2,013,347
Seoul, Gyeonggi-do, and Incheon	10,875	4068	37.4	38.2	5,638,653
20–24 years	1005	28	2.8		782,962
25–29 years	1706	75	4.4		961,554
30–34 years	2273	202	8.9		901,312
35–39 years	1864	496	26.6		909,856
40–44 years	2041	1387	68.0		1,037,250
45–49 years	1986	1880	94.7		1,045,720
Jeolla province	940	353	37.6	36.6	886,905
20–24 years	103	1	1.0		138,289
25–29 years	92	5	5.4		134,352
30–34 years	212	18	8.5		119,079
35–39 years	171	43	25.2		137,057
40–44 years	169	106	68.7		171,465
45–49 years	193	180	93.3		186,664
Chungcheong province	768	304	39.6	40.4	1,048,157
20–24 years	48	1	2.1		148,857
25–29 years	100	5	5.0		157,077
30–34 years	220	28	12.7		152,929
35–39 years	125	32	25.6		172,280
40–44 years	130	100	76.9		205,387
45–49 years	145	138	95.2		211,629
Gangwon-do	431	75	17.4	38.2	257,199
20–24 years	100	5	5.0		38,301
25–29 years	115	5	4.4		37,133
30–34 years	106	11	10.4		35,925
35–39 years	54	14	25.9		39,971
40–44 years	40	24	60.0		50,578
45–49 years	16	16	100.0		55,292
Gyeongsang province	337	171	50.7	43.1	2,311,737
20–24 years	24	1	4.2		323,212
25–29 years	36	2	5.6		341,687
30–34 years	63	10	15.9		325,970
35–39 years	55	19	34.6		369,443
40–44 years	76	57	75.0		464,466
45–49 years	83	82	98.8		486,960

^1^ Diminished ovarian reserve is defined by Korean guidelines for the infertility support program as serum AMH ≤ 1.0 ng/mL or serum FSH ≥ 12.0 mIU/mL.

## Data Availability

The datasets generated and analyzed during the current study are available from the corresponding authors on reasonable request.
